# Editorial: Updates on the Neuropathology of Sudden Unexplained Perinatal Death and Other Neurodevelopmental Disorders

**DOI:** 10.3389/fneur.2021.745860

**Published:** 2021-09-22

**Authors:** Anna Maria Lavezzi, Ana Paula Abdala, William P. Fifer

**Affiliations:** ^1^Lino Rossi Research Center, University of Milan, Milan, Italy; ^2^School of Physiology, Pharmacology & Neuroscience, Faculty of Life Sciences, University of Bristol, Bristol, United Kingdom; ^3^Departments of Psychiatry and Pediatrics, Columbia University, New York, NY, United States

**Keywords:** neuropathology, sudden perinatal death, brain dysfunctions, neurodevelopmental disorders, environmental risk factors

Perinatal mortality includes both fetal demises (stillbirths) and deaths in the first week of life. Worldwide, there are over 6.3 million perinatal deaths a year, almost all of which occur in developing countries (1). Stillbirths and neonatal deaths have many common determinants, such as maternal diseases, adverse prenatal exposure, inadequate care or complications during pregnancy and delivery, and genetic mutations. The first few hours of postnatal life are also particularly sensitive, as this is a critical time for a successful transition from intrauterine to extrauterine life wherein newborns are less responsive and more vulnerable to stressors. In case of sudden perinatal death, an important first step is the post-mortem examination since it can reveal the pathology underlying the possible causes of this inauspicious event (2). However, no unique etiology can be determined in most pre- and post-natal deaths, even after accurate autopsy investigations. Detailed examination of the autonomic nervous system can often reveal subtle developmental alterations, potentially providing a plausible explanation for sudden death (3, 4). Other neurodevelopmental disorders characterized by profound dysautonomia caused by single gene mutations may also lead to sudden unexplained death in perinatal life and infancy. Some of these syndromes are also thought to involve a state of “immaturity” of autonomic control systems. They include Rett syndrome, Cyclin-dependent kinase-like 5 (CDKL5) deficiency disorder, Pitt-Hopkins syndrome, Congenital central hypoventilation syndrome (CCHS), and GRIN1-Related Neurodevelopmental Disorder (5–9).

The primary rationale for this Research Topic has been to advance the state of knowledge and expertise for investigating the neuropathology of unexplained perinatal deaths and, in particular:

- contribute to the identification of the pathogenic mechanisms underlying these deaths (especially if known gene mutations are absent), in the context of interactions with environmental risk factors (e.g., early exposure to smoking, air and water pollution, pesticides, food contamination) and neuropathological findings.
- facilitate the development of evidence-based prevention and management strategies to decrease the incidence of these inexplicable and devastating deaths.

Another aim of this proposal was to deepen knowledge regarding developmental brain dysfunctions that can manifest later in life as neuropsychiatric symptoms, impaired motor function, learning, language, or non-verbal communication behaviors such as crying, or autism spectrum disorders (10–12). A related goal was to inform literature on the role of early life experiences, often associated with pre- or peri-natal environment exposures, in shaping the developing brain and vulnerability for the later neurodevelopmental outcome.

This Research Topic presents a series of articles and reviews written by leading authors and covering most aspects of state-of-the-art research on the neuropathology of fetal and infant sudden death and other neurodevelopmental disorders.

The papers are grouped into two main sections, according to the purposes of the Research Topic:

1) Neuropathology of Sudden Unexplained Perinatal Death
2) Neuropathology of Other Neurodevelopmental Disorders

**1) Neuropathology of Sudden Unexplained Perinatal Death**

The articles are distributed in the following sub-sections:

**(a) Neuropathological research on sudden infant death**

- Blackburn et al. present an epidemiological study on the neuropathology of sudden infant death syndrome (SIDS). These authors point out that the underlying death mechanism reported in many studies is highly controversial. Using machine learning tools, they emphasize the existence of three distinct groups of SIDS, each with a unique peak age of death, and specific epidemiological and extrinsic risk factors.
- McGuone et al. provide a review on Sudden Unexplained Death in Childhood (SUDC), i.e., the unexpected death of a child over 12 months of age that remains unexplained after a thorough case investigation. The authors emphasize the scarce and unsatisfactory nature of literature on this subject, especially on the neuropathology of SUDC, and advocate greater interdisciplinary participation in research efforts to elucidate the underlying mechanisms, especially to institute preventive strategies.

**(b) Neuropathology of neonatal breathing**

- A thorough description of the multiple systems and mechanisms that underlie regulation of breathing and cardiovascular processes in newborns is represented by the contribution of Harper and Kesavan. This interesting article reviews, among other topics, the rationale and empirical evidence for use of peripheral locomotor muscle pacing as an alternative intervention for disordered breathing, an often overlooked mechanism to stabilize breathing.

**(c) Genetics**

- Congenital central hypoventilation syndrome (CCHS) is a genetic neurodevelopmental disorder with an autosomal dominant transmission caused by heterozygous mutations in the *PHOX2B* gene (8). Bachetti et al. consider its similarity to an idiopathic apparent life-threatening event (IALTE) and sudden unexpected infant death (SUID/SIDS). The authors report, for the first time, on the genetic screening of the three exons of the PHOX2B gene in Italian IALTE and SUID/SIDS cases. The study finds a statistically significant association between common 3′UTR variants in the exon 3 of the PHOX2B gene with these two pathologies, suggesting that CCHS, ALTE, and SUID/SIDS might be members of the same group of respiratory autonomic disorders of infancy.
- Ke and Chen report a rare case of a heterozygous nonsense mutation in the *CTNNB1* gene in a 15-month-old girl with a complex phenotype (dysmorphic features, microcephaly, hypotonia, development delay, retinal detachment, and polydactyly) associated with a neurodevelopmental disorder with spastic diplegia and visual defects.

**(d) Immunohistochemical studies**

- Alwazzan, Mehboob, Hassan et al. analyze the immunoexpression of Neurokinin-1 receptor (NK-1R), a receptor of tachykinin peptide substance P (SP), in miscarriage occurring in the first trimester of pregnancy. The authors highlight the involvement of SP/NK-1 receptor system dysregulations in these deaths and suggest the use of NK-1R antagonists to diagnose and treat spontaneous abortion.
- Another immunohistochemical study by Alwazzan, Mehboob, Gilani et al. shows that the alpha 7-nicotinic acetylcholine receptors (α7-nAChR) are highly expressed in the placenta and products of conception during the first trimester. These receptors could then be involved in sudden fetal deaths and complications of pregnancy.

**(e) Perinatal environment exposure**

- The harmful effects of maternal tobacco smoke on the nervous system of fetuses and newborns are covered in the mini-review by Bednarczuk et al. The authors examine literature in this field, emphasizing the mechanisms by which smoking in pregnancy can cause brain abnormalities in sudden intrauterine unexplained death syndrome (SIUDS) and SIDS and the need to educate women on these harms.
- Lucchini et al. investigate the effects of chronic tobacco and alcohol consumption during pregnancy on autonomic function in a wide population of fetuses at ≥34 weeks gestational age, by quantifying heart rate, heart rate variability, movement, and heart rate-movement coupling. The results of this study contribute to identifying new biomarkers and understanding the mechanisms underlying risk for adverse outcomes.
- The serious consequences of cigarette smoke and alcohol absorption in pregnancy are also the focus of the contribution by Vivekanandarajah et al. Through a multicenter longitudinal study and by using autoradiographic methods to highlight the nicotinic receptor binding in the brainstems of infants dying of SIDS, the authors provide evidence that developmental factors paired with changes in nicotinic receptor binding are related to the cause of death as well as exposure to maternal cigarette smoking.

**2) Neuropathology of Other Neurodevelopmental Disorders**

- Rett syndrome is caused by the loss of function of the transcription factor methyl CpG-binding protein 2 (MeCP2) (13). In an experimental study, Ward et al. compare hypoxic ventilatory responses in mice with cell-type specific knockout or rescue of *Mecp2*. The study suggests that MeCP2 expression in excitatory, inhibitory, or dopaminergic/noradrenergic neural cells is essential for normal hypoxic ventilatory responses. The authors explore the implications of these findings for the pharmacological treatment of breathing abnormalities and understanding the pathophysiology of sudden death in Rett syndrome.
- Another interesting contribution on the Rett Syndrome is given by the systematic review article of Singh et al. This study, which focuses on the autonomic features of sudden unexpected death in pediatric epilepsy, offers interesting indications on the management of epilepsy in patients with Rett Syndrome.
- Congenital central hypoventilation syndrome (CCHS) is the focus of the review article by Di Lascio et al. The study analyses various *in vivo* and *in vitro* approaches aimed at better understanding the CCHS molecular pathogenetic mechanism, in order to reduce the damage caused by the aberrant function of mutant PHOX2B and provide new therapeutic strategies.
- New insights into hypoxic ischemic encephalopathy (HIE) of the full-term newborn derive from the experimental study by Gotchac et al. The authors develop a new rodent model, using acute hypoxic cardiac arrest to induce HIE-like injury in post-natal rats. The study, which combines radiological, histological, and behavioral testing, finds that neurological changes that persist into adulthood, suggesting that the model may recapitulate milder forms of HIE. This model may be useful for understanding the long-term neurological and psychiatric consequences of oxygen deprivation during perinatal life.

Together, these publications expand current knowledge on the neuropathology of sudden unexplained perinatal/infant death and other neurodevelopmental disorders, such as the Rett syndrome and CCHS, thus allowing the broadening of the diagnostic criteria and preventive strategies. As editors of this Research Topic, we express our sincere gratitude to the authors who accepted the invitation to participate and for their significant efforts in identifying interesting approaches that explain the pathogenesis of these conditions. We would also like to thank the reviewers for their significant comments, elevating the quality of the submitted articles. Finally, we thank Frontiers and, in particular, the editorial office for essential support.

## Author Contributions

AL, AA, and WF: the editors of this Research Topic, together wrote the editorial that summarizes the contents of all the 13 published articles. All authors contributed to the article and approved the submitted version.

## Funding

AA was funded by the International Rett Syndrome Foundation (#3803).

## Conflict of Interest

The authors declare that the research was conducted in the absence of any commercial or financial relationships that could be construed as a potential conflict of interest.

## Publisher's Note

All claims expressed in this article are solely those of the authors and do not necessarily represent those of their affiliated organizations, or those of the publisher, the editors and the reviewers. Any product that may be evaluated in this article, or claim that may be made by its manufacturer, is not guaranteed or endorsed by the publisher.

## References

[1] World Health Organization. Neonatal and Perinatal Mortality: Country, Regional and Global Estimate. World Health Organization (2006). Available online at: https://apps.who.int/iris/handle/10665/43444

[2] Sorop-Florea M Ciurea RN Ioana M Stepan AE Stoica GA Tănase F. The importance of perinatal autopsy. Review of the literature and series of cases. Rom J Morphol Embryol. (2017) 58:323–37. 28730216

[3] Bright FM Vink R Byard RW. Brainstem neuropathology in sudden infant death syndrome. In: Duncan JR Byard RW, editors. SIDS Sudden Infant and Early Childhood Death: The Past, the Present and the Future. Adelaide, SA: University of Adelaide Press (2018) 589–614. 30035961

[4] Grafe MR Kinney HC. Neuropathology associated with stillbirth. Semin Perinatol. (2002) 26:83–8. 10.1053/sper.2002.29862 11876572

[5] Julu PO Kerr AM Hansen S Apartopoulos F Jamal GA. Functional evidence of brain stem immaturity in Rett syndrome. Eur Child Adolesc Psychiatry. (1997) 6(Suppl. 1):47–54. 9452920

[6] Olson HE Demarest ST Pestana-Knight EM Swanson LC Iqbal S Lal D. Cyclin-dependent kinase-like 5 deficiency disorder: clinical review. Pediatr Neurol. (2019) 97:18–25. 10.1016/j.pediatrneurol.2019.02.015 30928302 PMC7120929

[7] Marangi G Ricciardi S Orteschi D Lattante S Murdolo M Dallapiccola B. The Pitt-Hopkins syndrome: report of 16 new patients and clinical diagnostic criteria. Am J Med Genet A. (2011) 155A:1536–45. 10.1002/ajmg.a.34070 21671391

[8] Moreira TS Takakura AC Czeisler C Otero JJ. Respiratory and autonomic dysfunction in congenital central hypoventilation syndrome. J Neurophysiol. (2016) 116:742–52. 10.1152/jn.00026.2016 27226447 PMC6208311

[9] Platzer K Lemke JR. *GRIN1*-related neurodevelopmental disorder. In: Adam MP Ardinger HH Pagon RA Wallace SE Bean LJH Mirzaa G, editors. GeneReviews®. Seattle, WA: University of Washington (2021). p. 1993–2021. 31219694

[10] Ogundele MO. Behavioural and emotional disorders in childhood: a brief overview for paediatricians. World J Clin Pediatr. (2018) 7:9–26. 10.5409/wjcp.v7.i1.9 29456928 PMC5803568

[11] Nudel R Christiani CAJ Ohland J Uddin MJ Hemager N Ellersgaard DV. Language deficits in specific language impairment, attention deficit/hyperactivity disorder, and autism spectrum disorder: an analysis of polygenic risk. Autism Res. (2020) 13:369–81. 10.1002/aur.2211 31577390 PMC7078922

[12] T-Pederson C Reisert H Adesman A. Wandering behavior in children with autism spectrum disorder and other developmental disabilities. Curr Opin Pediatr. (2021) 33:464–70. 10.1097/MOP.0000000000001038 34226426

[13] Monteggia LM Kavalali ET. Rett syndrome and the impact of MeCP2 associated transcriptional mechanisms on neurotransmission. Biol Psychiatry. (2009) 65:204–10. 10.1016/j.biopsych.2008.10.036 19058783 PMC3001289

